# High Levels of Anxiety, Depression, Risk of Suicide, and Implications for Treatment in Patients with Lamellar Ichthyosis

**DOI:** 10.3390/healthcare11142071

**Published:** 2023-07-20

**Authors:** Hernán Cortés, Lizbeth Cariño-Calvo, Octavio D. Reyes-Hernández, Martín Rojas-Márquez, Jonathan J. Magaña, Pablo A. Vizcaino-Dorado, Edgar Y. Villegas-Vazquez, Laura Itzel Quintas-Granados, Elizabeth Jiménez-Islas, Valeria A. Cortés-Mollinedo, Gerardo Leyva-Gómez, Manuel González-Del Carmen

**Affiliations:** 1 Laboratorio de Medicina Genómica, Departamento de Genómica, Instituto Nacional de Rehabilitación Luis Guillermo Ibarra Ibarra, Ciudad de México 14389, Mexico; 2 Facultad de Ciencias Químicas, Universidad Veracruzana, Orizaba 94340, Mexico; 3 Laboratorio de Biología Molecular del Cáncer, UMIEZ, FES Zaragoza, Universidad Nacional Autónoma de México, Ciudad de México 09230, Mexico; 4 Hospital Psiquiátrico Infantil Dr. Juan N. Navarro, Secretaría de Salud, Ciudad de México 14080, Mexico; 5 Laboratorio de Farmacogenética, UMIEZ, FES Zaragoza, Universidad Nacional Autónoma de México, Ciudad de México 09230, Mexico; 6 Unidad de Estudios Superiores Tultitlán, Universidad Mexiquense del Bicentenario, Ocoyoacac 54910, Mexico; 7 Departamento de Farmacia, Facultad de Química, Universidad Nacional Autónoma de México, Ciudad de México 04510, Mexico; 8 Facultad de Medicina, Universidad Veracruzana, Ciudad Mendoza 94740, Mexico

**Keywords:** lamellar ichthyosis, anxiety, depression, suicide risk

## Abstract

Lamellar ichthyosis (LI) is a genodermatosis that injures the structure and function of the skin, affecting the appearance and self-esteem of patients, which may seriously impair their mental health and quality of life. In the present study, we determined anxiety, depression, and suicidal risk levels in patients with LI through the Beck anxiety and depression inventories (BAI and DBI-II, respectively) and the SAD PERSONS scale (SPS). We observed that anxiety, depression, and suicidal ideation were strongly associated with the LI (Cramér’s V = 0.429, 0.594, and 0.462, respectively). Furthermore, patients with LI showed a significant increase in the scores of anxiety, depression, and suicidal risk (*p* = 0.011, <0.001, and 0.001, respectively) compared to individuals without the disease. Additionally, the suicide risk increased even more in patients who presented comorbidity of anxiety and depression than in patients who presented only anxiety or depression (*p* = 0.02). Similarly, the increase in the BAI scores correlated with the score observed on the SPS. Our results indicate that patients with LI have higher levels of anxiety and depression compared to individuals without the disease, which could be associated with suicidal risk. Therefore, the collaborative involvement of skin and mental health professionals is necessary to manage patients with LI appropriately. We believe that psychiatric studies and individual evaluations must be performed in LI patients to determine a treatment that, in addition to reducing skin symptoms, focuses on reducing the levels of depression and anxiety and improving the quality of life to reduce the risk of suicide.

## 1. Introduction

Inherited ichthyoses are a group of skin disorders with different causes and levels of severity. They can be divided into two main types: syndromic and non-syndromic ichthyoses [1]. Syndromic ichthyoses involve other problems besides the skin, such as neurological, muscular, or endocrine abnormalities. Some examples of these rare syndromes are Sjögren–Larsson syndrome and Netherton syndrome. Non-syndromic ichthyoses are more common and only affect the skin [2]. The main types are recessive X-linked ichthyosis (RXLI), ichthyosis vulgaris (IV), keratinopathic ichthyosis (KPI), and autosomal recessive congenital ichthyosis (ARCI). These disorders are usually caused by mutations in genes that have essential roles in various skin functions, such as the synthesis of lipids, regular desquamation, and repairing DNA. These genetic variants can result in problems such as a broken stratum corneum, a damaged skin barrier, and a higher water loss through the skin, leading to different clinical features [1].

Lamellar ichthyosis (LI) belongs to the so-called ARCI, among which are also other severe presentations such as harlequin ichthyosis (HI) and congenital ichthyosiform erythroderma (CIE) [1]. LI is a genodermatosis characterized by thick brown plate-shaped scales distributed throughout the body surface of patients. Likewise, IL patients suffer from excessive dryness, hyperkeratosis, pruritus, skin scaling, inflammation, fissures, and pain, which seriously affects the structure and protective function of the skin, increasing the susceptibility to varied cutaneous infections [1,2]. Furthermore, severely affected patients can suffer from eclabium and ectropion. Despite being classified as a rare disease with a worldwide prevalence between 1:200,000–1:300,000, higher prevalence has been reported in Galicia (1:33,000) and several Mexican rural populations (1:1348) as a consequence of the existence of founder mutations [3,4,5,6]. In these Mexican communities, severe skin damage has been observed according to the Congenital Ichthyoses Severity Index (CISI), strongly influenced by the lack of care and treatment [3].

Skin diseases that affect the face and noticeable body parts are usually associated with damage to mental health, which would lead to mood problems, even affecting social aspects such as work and education of patients. In this respect, LI may significantly affect the physical appearance of the patients, which can produce a loss of confidence, shame, shyness, low self-esteem, social isolation, and discrimination, with emotional troubles and mental health diseases. Although the presence of mental disorders such as anxiety, depression, and suicidal ideation in patients with severe skin diseases has been clearly established, there is a notable lack of knowledge about these disorders in patients with LI. Thus, it is necessary to identify if patients with LI suffer from these conditions (that are not easily detectable in the initial diagnosis) to develop new approaches and treatments to improve their quality of life [7,8,9].

Anxiety is the most common mental disorder worldwide; it is estimated that more than 30% of the world population has experienced anxiety-related symptoms in their lifetime, including irritability, tachycardia, muscle tension, and diaphoresis. In many cases, the symptoms can be exacerbated and present excessive worry that can be accompanied by cognitive and physical symptoms such as fatigue, lack of concentration, and sleep problems, giving rise to generalized anxiety disorder (GAD) [10]. Similarly, depression is a highly prevalent mental disease that occurs in about 30% of illnesses associated with the skin and is characterized by feelings such as loss of energy, guilt, social isolation, anhedonia, and sadness, which can even lead to loss of pleasure in life. In this regard, depression is often a factor strongly associated with the severity of the dermatological disease [11]. It has been suggested that these mental conditions that result in affective disorders could be associated with the occurrence of suicide risk [12,13]. Suicide is one of psychiatry’s primary concerns and a prominent cause of death worldwide [14]. It is related to a process involving weariness of life, death wishes, and suicide ideation and attempt, culminating in the patient’s death. It is estimated that for every suicide committed, there are at least ten suicide attempts in which sociodemographic and family factors intervene, as well as mental conditions or diseases that affect the appearance of individuals [15].

The prevalence of anxiety, depression, suicidal Ideation, and suicide attempts in patients with dermatological disorders such as acne, psoriasis, and atopic dermatitis (AD) is higher than in the general population [16,17,18]. Therefore, we hypothesized that patients with LI, where skin damage may be more severe and evident, could present those mental disorders. In this regard, we recently described communities with a high prevalence of LI; patients show severe disease manifestations and mental conditions that affect their quality of life [3,19]. Thus, we decided to investigate whether these patients suffer from anxiety, depression, and suicidal ideation to generate information that can help establish strategies and interventions to improve their overall health.

## 2. Materials and Methods

Our study included 24 patients ≥ 17 years old with a clinical and genetic diagnosis of LI, and all of them belong to three municipalities of the High Mountains Region of Veracruz State previously described [3]. As a control group, we recruited 24 sex-matched healthy individuals of similar ages from the same communities without ichthyosis or other skin disease. The control group comprised relatives and unrelated individuals who accompanied the LI patients during social assistance carried out by civil associations “Ichthyosis Mexico” and “Genes Latino America”. We tested age differences between the LI and control groups by statistical analysis through the Student *t*-test (*p* = 0.205). All the participants signed informed consent before participating in our study and agreed to answer the surveys. The Research and Bioethics Committee of the Universidad Veracruzana approved our research protocol (020-2021-FMCM-CI-CEI). We carried out all the procedures of this research according to the Code of Ethics of the Declaration of Helsinki.

We used the Beck Anxiety Inventory (BAI) and the Beck Depression Inventory-II (DBI-II) to investigate the anxiety and depression levels in the participants, respectively. Both scales use 21 items with a score of 0–3 (no symptoms to symptoms strongly present), which allows classifying four severity levels according to the global score. Total scores to determine the level of anxiety were; no anxiety or minimal (0–7), mild (8–15), moderate (16–25), and severe (26–63). The levels of depression that can be obtained are: no depression or minimal (0–13), mild (14–19), moderate (20–28) and severe (29–63) [19,20,21]. The determination of suicide risk was carried out under the supervision of a psychiatrist (MRM) by using the SAD PERSONS scale (SPS), a tool widely used in clinical practice and psychiatry. This scale is a 10-item mnemonic device in which several suicide risk factors are represented with letters (S = sex, A = age, D = depression, *p* = previous attempts or psychiatric care, E = excessive alcohol or drug use, R = rational thinking loss, S = separated/divorced/widowed, O = organized or serious attempt, N = no social supports, S = stated future intent). Each factor has the same value assigning 1 when present and 0 when absent, so a higher score represents a higher risk of suicide [22,23,24]. We measured the clinical severity according to the CISI, which comprises the variables squamae (score 1–5), erythema (score 1–6), and alopecia (score 1–5). Higher scores indicate worsening symptoms [25]. All these tools were applied by trained personnel.

As indicated in figures and tables, variables were expressed as percentages, medians, and ranges. The normality of variables was assessed by the Shapiro–Wilk test and analyzed using Fisher’s exact test, Mann–Whitney U test, and Spearman correlation. We calculated the measure of association by Cramér’s V coefficient. To calculate the odds ratio (OR, 95% confidence interval), the different mental health variables were dichotomized as follows; no depression (minimal depression) vs. presence of depression (mild + moderate + severe); no anxiety (minimum anxiety) vs. presence of anxiety (mild + moderate + severe); no suicide risk (minimum suicide risk) vs. presence of suicide risk (mild + moderate + severe). *p* values < 0.05 were considered statistically significant. Data were analyzed using SPSS software version 25.

## 3. Results

**Figure 1:**
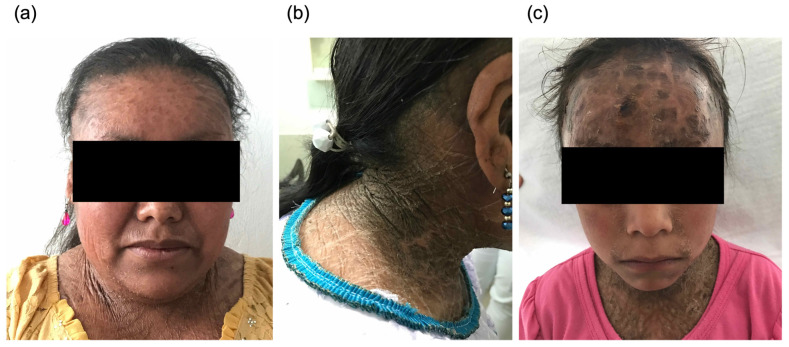
Patients with lamellar ichthyosis. Brown scales are present in large zones of their body, including the face (**a**,**c**) and neck (**b**).

The study population consisted of 24 patients with LI, all of them carrying the homozygous mutation c.1054C > G [P.Pro352Ala] previously described, as well as 24 healthy individuals [3]. The studied patients’ epidemiological and genetic aspects and geographic location were previously described [3,19,26]. The average age was 31 ± 9 years old (range 17–52 years), of which 42% (n = 10) were men and 58% (n = 14) were women. All the patients exhibited a severe level of squamae according to CISI and a pattern of dark scales in most of the body, including the face and neck (Figure 1). Other frequent clinical characteristics in most patients were collodion membrane observed at birth, palmoplantar hyperkeratosis, palmoplantar hyperlinearity, hypohidrosis, pruritus, ectropion, and scarring alopecia. In contrast, in less than 30% of the patients, subungual hyperkeratosis, onychogryphosis, nose and ear cartilage malformation, and keratosis pilaris were observed (Table 1). Interestingly, many patients presented digital contractures, which could limit the ability to manipulate utensils in daily life. In the control group, all the mentioned symptoms were absent, the average age was 35 ± 12 years old (range 19–55 years), and 42% (n = 10) of participants were men and 58% (n = 14) were women.

The anxiety levels measured by the BAI were the following: in patients with LI, 8% presented a minimum level, 42% showed a mild level, 33% exhibited moderate anxiety, and 17% showed severe anxiety. In contrast, in the control group, the highest percentage (46%) did not present anxiety (minimum level), while 21% had a mild level, 25% had moderate anxiety, and only 8% showed a severe level. Regarding the levels of depression in LI patients, 20% did not present depression, 20% presented mild depression, and for moderate and severe depression, 30% were found at each level. Regarding the healthy volunteers, 79% had no depression, 4% had mild depression, 13% presented moderate depression, and 4% showed severe depression (Table 2).

Regarding the suicidal risk analyzed in patients with LI, 29% did not present risk, while the highest percentage (37%) showed a mild risk, 30% exhibited a medium risk, and 4% had a high risk. In the control group, we observed that most participants did not present suicide risk (70%), 25% had a mild risk, and only one individual (4%) exhibited a medium risk. No members from the control group presented a high risk of suicide. Cramér’s V association coefficient obtained in the three cases showed a significant association of ichthyosis with anxiety, depression, and risk of suicide (Table 2).

Additionally, we compared the medians of the global BAI, DBI-II, and SPS scores between the patients with LI and the group of healthy volunteers. We found significantly higher median values of the global scores for the three scales in the patients with LI (Table 3). The higher difference in the medians was observed in the comparison of the DBI-II (25 in IL patients vs. 8.5 in control patients). The OR were 9.3, 14.4, and 5.8 for anxiety, depression, and suicidal risk, respectively (Table 3). Interestingly, the median of the global scores of the SPS was higher in patients whose results indicated the simultaneous presence of anxiety and depression (median = 4) compared to patients with only anxiety or only depression (median = 2) (Table 4), which suggests a synergistic effect between anxiety and depression to increase the risk of suicide (OR = 18.7). We did not find a significant difference between men and women in the global scores of anxiety, depression, and suicide risk in LI patients (*p* = 0.681, 0.447, and 0.841, respectively).

**Figure 2:**
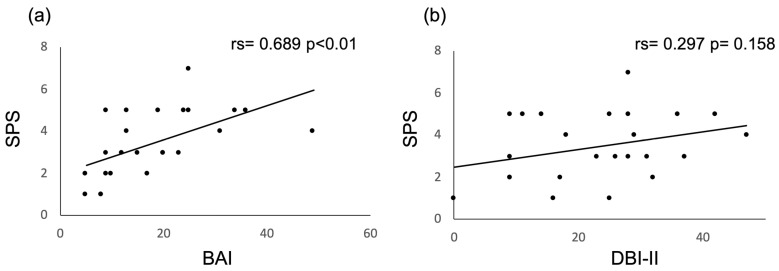
Correlation among BAI, DBI-II, and SPS scores in LI patients. (**a**) Correlation between the risk of suicide and anxiety scores. (**b**) Correlation between the risk of suicide and depression scores. rs = Spearman’s correlation coefficient.

On the other hand, we performed a correlation analysis by comparing the global scores of the SPS with the BAI and DBI-II scores in patients with LI and control individuals. We found that the score obtained on the BAI had a strong correlation (rs = 0.689, *p* < 0.01) with the SPS score (Figure 2a), while the score referring to depression had a weak and not significant correlation with the level of suicidal risk (rs = 0.297, *p* = 0.158) (Figure 2b). In the control group, no significant correlation was observed between anxiety and suicidal risk (rs = −0.169, *p* = 0.429) or depression and suicidal risk (rs = 0.238, *p*= 0.263). Additionally, the age of IL patients strongly correlated with the overall DBI-II score (rs = 0.52, *p* < 0.01), which was not observed against anxiety (rs = 0.1, *p* = 0.63) or suicidal risk (rs = 0.2, *p* = 0.335).

## 4. Discussion

LI affects the whole body of patients, presenting a large number of brown scales in visible areas, and it is accompanied by itching, pain, and scaling, among other symptoms. In many cases, the severity of the symptoms is increased due to factors such as the lack of treatment and environmental and socioeconomic conditions. In these cases, many patients show impairment in self-perception and mood. Previous studies have shown that some skin diseases can significantly influence the mental health and quality of life of affected patients with a strong emotional and psychological impact that even affects aspects of the patient’s social life. This impact is generally reflected in the presence of anxiety and depression [7,19]. We have previously reported a population with a high prevalence of LI characterized by severe symptoms that dramatically affect patients’ physical appearance, confidence, and self-esteem, causing mental alterations and impairment in their quality of life, possibly associated with suicide ideation [7,19,27]. Suicide is usually preceded by suicidal ideation and behavior and is generally related to psychiatric disorders and emotional damage. Thus, identifying individuals with these characteristics could help to create strategies to prevent this fatal outcome [28]. In this study, we explored the presence of anxiety and depression in patients with severe symptoms of LI. Since these mental disorders can be associated with suicidal ideation, we also decided to investigate suicide risk levels.

Suicide is a public preventable health problem worldwide. In the United States, suicide is a significant cause of death in adolescents and young adults (10–34 years old, the second leading cause of death). Although actions have been carried out to reduce aspects such as suicidal ideation and suicide attempts, the prevalence has not changed in recent years. In many cases, identifying factors such as tolerance to physical pain and low fear of death can indicate suicidal risk. Other elements of great importance that influence the presence of suicide include aspects such as psychiatric and emotional disorders, as well as the presence of interpersonal violence. In this respect, studies evaluating suicidal risk should be carried out on people who suffer from some psychiatric comorbidity, exacerbated chronic diseases, or dermatological diseases with facial lesions that can lead to the social exclusion of the patient, among others [28,29]. The last is observed in patients with LI, whose scales cover most of their body, including the face, scalp, and neck. These patients may suffer stigmatization, isolation, and hopelessness [3,19,27]. Several reports indicate that depression strongly correlates to suicidal ideation and risk [13]. Interestingly, we observed higher anxiety, depression, and suicidal risk levels in patients with LI compared to the control group (OR = 9.3, 14.4, and 5.89, respectively). It has been proposed that aspects related to suicide are associated with mood, psychotic, personality, and anxiety disorders and that the presence of two or more of those disorders could contribute to an increase in the manifestation of suicidal ideation and risk. In this study, we found that the simultaneous occurrence of anxiety and depression increases the median of SPS global scores, which represents an additional risk of suicide compared to patients with only anxiety or depression (OR = 18.75).

Regarding other skin diseases, suicidal ideation in patients with AD increases compared to those without the disease (OR = 4.32). It is also associated with the severity of the disease, increasing the need to use anxiolytic drugs and antidepressants [30,31]. The presence of AD, in addition to increasing the level of suicidal ideation, also increased the number of suicide attempts compared to the general population [18]. Similarly, some studies reported that 8.6–12.9% of patients with acne showed suicidal ideation associated with depression, anxiety, and damage to quality of life. In the case of children with skin diseases (acne, AD, and psoriasis), suicidal ideation was associated with depression, stigmatization, and bullying [32]. However, these studies’ limitation is that suicidal ideation is evaluated only through one item, which might give subjective results [31,33,34,35]. In this context, age could be associated with the severity of mental damage in patients with dermatological diseases. In our study, we observed a strong correlation between age and the level of depression, probably because older people could easily perceive social problems as bullying and stigmatization [32]. Hidradenitis suppurativa has also been associated with depression, anxiety, and impaired quality of life [36]. Furthermore, patients with hidradenitis suppurativa exhibit an increased risk of completed suicide (1.4/1000) compared to the general population (0.66/1000) [37]. In contrast to the amount of information available on mental disorders and suicidal risk in AD, acne, psoriasis, and hidradenitis suppurativa, according to our knowledge, this is the first study that describes the presence of suicidal risk in patients with severe LI. In this regard, we are aware that the sample size of this study is relatively small, which could make it difficult to interpret our results. However, we should note that this study involved patients with the same genetic mutation and living in the same communities, making them suitable for assessing any clinical aspect. Moreover, we compared them with a group of healthy volunteers of similar age and sex from the same region, which helped us rule out the potential negative influence of factors such as environment, society, and economy on mental health (possible confounding factors). Therefore, we think this cohort of patients is suitable for future comparative analyses with patients of different geographical origins and to complete this disease’s clinical and molecular characterization.

On the other hand, there are no reports on molecular mechanisms that could associate the presence of lamellar ichthyosis with any mental illness. However, in patients with X-linked ichthyosis, it has been described that the lack of expression of the enzyme steroid sulfatase is associated with variations in brain activity leading to changes in the patient’s personality and mood, depression, disruptive behavior, attention deficit, and hyperactivity, although the substrates that could mediate such behaviors are unknown [38]. Mutations in TGM1, Ichthyin, ALOXE3/12B, FLJ39501, and ABCA12 are frequently associated with lamellar ichthyosis. The consequences of these mutations could imply some interaction with the mechanisms that lead to the appearance of depression, including changes in the secretion of hormones, interleukins, or the activation of protein kinases [1,39]. Likewise, from a physiological and embryologic point of view, the skin and the brain are closely linked since they derive from the same germ layer, suggesting a possible functional connection [27]. Hence, we can speculate that severe injury to the skin could be physiologically related to developing mental health problems such as generalized anxiety disorder and depression. However, further molecular studies are necessary to understand these mechanisms.

On the other hand, treatments for IL should be applied throughout the patient’s life to reduce the symptoms present daily, such as dryness, excess scales, itching, and pain, through topical emollients and keratolytic agents. Likewise, it is well known that retinoids are an excellent option for treating ichthyosis. However, even though patients improve significantly the structure and functionality of their skin, retinoids such as tretinoin, adapalene, isotretinoin, and acitretin should be used in a controlled way because they can generate side effects such as excessive dryness, redness, ocular pruritus. Furthermore, retinoids can cause more severe consequences, such as damage to bone development, photophobia, eye pain, adverse effects on liver function, and even damage to fetal development when applied during pregnancy [40,41]. Likewise, adverse mental health effects have been reported in patients treated with retinoids. Nonetheless, these results may be controversial since it also has been reported that in addition to decreasing the severity of the symptoms, the use of isotretinoin was also associated with a decrease in anxiety, depression, and sleep problems, which led to reduced psychotropic medication use in patients with acne. Thus, the effects could depend on diverse factors, including dosage, use time, and dermatological disease severity [42,43]. Some alternatives to reduce the side effects of retinoids would be utilizing drug delivery mechanisms, including nanoparticles, which could imply a more specific therapeutic effect with lower doses [44]. Systems such as liposomes or lipid nanoparticles smaller than 100 nm can be used, even in topical applications, to carry out a local action below the stratum corneum. These systems could facilitate the distribution of drugs that are even poorly permeable, which is essential for the successful treatment of dermatological diseases, reducing the risk of absorption and the presence of adverse effects [45]. In patients with acne vulgaris, a drug delivery system containing tretinoin has been used, which has proven to be more effective than the conventional 0.05% tretinoin cream without reaching toxic concentrations of the drug in plasma [46]. A topical isotretinoin formulation has recently been developed, which increased hydration levels and favored the reduction of scale severity in patients with congenital ichthyosis and which could also have less risk of presenting side effects without altering laboratory parameters [47]. This approach could be used in patients with IL to improve skin symptoms significantly, and because there is a greater risk of depression and impaired quality of life in patients with moderate and severe symptoms compared to patients with minimal or mild scaling, improvement in skin symptoms would be expected to correlate with decreased damage at the mental health [19]. Subsequent studies of the patients are necessary to evaluate the improvement of the symptoms using effective treatments and their correlation with improving mental health, covering aspects such as anxiety, depression, suicidal risk, and quality of life.

Thus, we must highlight the importance of a multidisciplinary approach in the mental care of patients with this disease. It is timely to mention that despite the availability of diverse questionnaires and self-applicable scales to measure aspects related to suicidal risk, none provides sufficient certainty to predict which individual will commit suicide. However, we think that our results can be interpreted carefully with the help of experts in dermatology and psychiatry, which can allow the creation of strategies that reduce the severity of the scales with an adequate treatment based on moisturizers, emollients, and keratolytic agents. Additionally, mental health monitoring is necessary, which can be quickly addressed through instruments designed to detect and quantify possible mental disorders such as depression, anxiety, and suicide risk. The information generated during the interview with the patients will allow the creation of specific treatment strategies that involve psychotherapeutic techniques such as cognitive behavior therapy and interpersonal therapy to increase social inclusion and overcome the mental disorders detected [48]. In cases where the duration of the symptoms is longer and where more significant damage is observed, the intervention of a psychiatrist will be necessary, evaluating the use of antidepressants and antianxiety drugs and considering psychiatric hospitalization. In addition to the above, social strategies should be carried out that involve individuals who cohabit with the affected population to reduce stigmatization and discrimination. Therefore, we believe that psychiatric studies and individual evaluations must be performed in LI patients to determine a treatment that, in addition to reducing skin symptoms, focuses on reducing the levels of depression and anxiety and improving the quality of life to reduce the risk of suicide.

## 5. Conclusions

In this article, we examined the prevalence and correlates of suicidal ideation, anxiety, and depression in patients with LI using a cross-sectional survey design and standardized instruments. The main findings were that patients with LI have higher levels of depression, anxiety, and suicidal ideation than the general population. This finding is consistent with other studies that have reported a significant psychological burden of skin diseases. These results highlight the need for comprehensive assessment and care of the mental health of patients with LI (similar to other skin diseases) and the importance of addressing the psychosocial factors that may contribute to their distress. Further research is needed to explore the mechanisms and mediators of the relationship between LI and suicidal ideation and to evaluate the effectiveness of interventions to prevent and reduce suicide risk in these patients.

## Figures and Tables

**Table 1:** Clinical characteristics of patients included in the study.

Clinical Features	n (%)
Collodion membrane ^a^	24 (100)
Brown plate-like scales ^b^	24 (100)
Palmoplantar hyperkeratosis ^b^	24 (100)
Palmoplantar hyperlinearity ^b^	21 (87)
Hypohidrosis ^a^	21 (87)
Pruritus ^a^	21 (87)
Ectropion ^b^	20 (83)
Scarring alopecia ^b^	19 (79)
Digital contractures ^b^	15 (62)
Subungual hyperkeratosis ^b^	7 (29)
Onychogryphosis ^b^	4 (16)
Malformation of the nose and ear cartilage ^b^	2 (8)
Keratosis pilaris ^b^	2 (8)

^a^ Interrogation; ^b^ Clinical examinations.

**Table 2:** Levels of anxiety, depression, and suicidal risk in patients with lamellar ichthyosis (LI) and control individuals according to BAI, DBI-II, and SAD PERSONS scales.

	N (%)					*p*-Value *	Cramer’s V
**Anxiety**							
		Minimum	Mild	Moderate	Severe		
LI	24 (100)	2 (8)	10 (41)	8 (33)	4 (16)	0.028	0.429
Control	24 (100)	11 (45)	5 (20)	6 (25)	2 (8)		
**Depression**							
		Minimum	Mild	Moderate	Severe		
LI	24 (100)	5 (20)	5 (20)	7 (29)	7 (29)	0.001	0.594
Control	24 (100)	19 (79)	1 (4)	3 (12)	1 (4)		
**Suicide risk**							
		No risk	Low	Medium	High		
LI	24 (100)	7 (29)	9 (37)	7 (29)	1 (4)	0.011	0.462
Control	24 (100)	17 (70)	6 (25)	1 (4)	0 (0)		

* *p*-value: Fisher exact test.

**Table 3:** Comparison of BAI, DBI-II, and SAD PERSONS scores between LI patients and control individuals.

	N	Median ^a^ (Range)	*p*-Value ^b^	OR ^c^
**BAI**				
LI	24	16 (5–49)	0.011	9.3 (1.778, 48.723)
Control	24	10 (0–34)		
**DBI-II**				
LI	24	25 (0–47)	<0.001	14.4 (3.58, 58.15)
Control	24	8.5 (2–33)		
**SAD PERSONS**				
LI	24	3 (1–7)	0.001	5.8 (1.699, 20.48)
Control	24	2 (0–6)		

^a^ Median of scores ^b^ *p*-value: U de Mann–Whitney ^c^ OR 95% confidence interval.

**Table 4:** Effect of comorbidity of anxiety and depression on SAD PERSONS scores in LI patients.

	N	Median ^a^ (Range)	*p*-Value ^b^	OR ^c^
LI				
Comorbidity of anxiety and depression	17	4 (1–7)	0.02	18.7 (2.06, 170.2)
Only depression or anxiety	7	2 (1–5)		

^a^ Median of SAD PERSONS scores; ^b^ *p*-value: U de Mann–Whitney; ^c^ OR 95% confidence interval.

## Data Availability

The data that support the findings of this study are available from the corresponding author upon reasonable request.

## References

[1] Vahlquist A., Gånemo A., Virtanen M. (2008). Congenital ichthyosis: An overview of current and emerging therapies. Acta Derm. Venereol..

[2] Schmuth M., Gruber R., Elias P.M., Williams M.L. (2007). Ichthyosis update: Towards a function-driven model of pathogenesis of the disorders of cornification and the role of corneocyte proteins in these disorders. Adv. Dermatol..

[3] González-Del Carmen M., Montaño S., Reyes-Hernández O.D., Vizcaíno-Dorado P.A., Leyva-García N., Morales-Morfín J.C., Diaz-Beltran W., Quinto-Santiago E., Msc L.C., Magaña J.J. (2020). High prevalence of autosomal recessive congenital ichthyosis in a Mexican population caused by a new mutation in the TGM1 gene: Epidemiological evidence of a founder effect. Int. J. Dermatol..

[4] Rodríguez-Pazos L., Ginarte M., Fachal L., Toribio J., Carracedo A., Vega A. (2011). Analysis of TGM1, ALOX12B, ALOXE3, NIPAL4 and CYP4F22 in autosomal recessive congenital ichthyosis from Galicia (NW Spain): Evidence of founder effects. Br. J. Dermatol..

[5] Hernández-Martín A., Garcia-Doval I., Aranegui B., De Unamuno P., Rodríguez-Pazos L., González-Enseñat M.A., Vicente A., Martín-Santiago A., Garcia-Bravo B., Feito M. (2012). Prevalence of autosomal recessive congenital ichthyosis: A population-based study using the capture-recapture method in Spain. J. Am. Acad. Dermatol..

[6] Kurosawa M., Uehara R., Takagi A., Aoyama Y., Iwatsuki K., Amagai M., Nagai M., Nakamura Y., Inaba Y., Yokoyama K. (2019). Results of a nationwide epidemiologic survey of autosomal recessive congenital ichthyosis and ichthyosis syndromes in Japan. J. Am. Acad. Dermatol..

[7] Guo F., Yu Q., Liu Z., Zhang C., Li P., Xu Y., Zuo Y., Zhang G., Li Y., Liu H. (2020). Evaluation of life quality, anxiety, and depression in patients with skin diseases. Medicine.

[8] Sun Q., Ren I., Zaki T., Maciejewski K., Choate K. (2020). Ichthyosis affects mental health in adults and children: A cross-sectional study. J. Am. Acad. Dermatol..

[9] Wren G.H., Humby T., Thompson A.R., Davies W. (2022). Mood symptoms, neurodevelopmental traits, and their contributory factors in X-linked ichthyosis, ichthyosis vulgaris and psoriasis. Clin. Exp. Dermatol..

[10] Showraki M., Showraki T., Brown K. (2020). Generalized Anxiety Disorder: Revisited. Psychiatr. Q..

[11] Fried R.G., Gupta M.A., Gupta A.K. (2005). Depression and skin disease. Dermatol. Clin..

[12] Bentley K.H., Franklin J.C., Ribeiro J.D., Kleiman E.M., Fox K.R., Nock M.K. (2016). Anxiety and its disorders as risk factors for suicidal thoughts and behaviors: A meta-analytic review. Clin. Psychol. Rev..

[13] Norton P.J., Temple S.R., Pettit J.W. (2008). Suicidal ideation and anxiety disorders: Elevated risk or artifact of comorbid depression?. J. Behav. Ther. Exp. Psychiatry.

[14] (2021). World Health Organization Suicide: Key Facts. https://www.who.int/news-room/fact-sheets/detail/suicide.

[15] Picardi A., Lega I., Tarolla E. (2013). Suicide risk in skin disorders. Clin. Dermatol..

[16] Dieris-Hirche J., Gieler U., Petrak F., Milch W., te Wildt B., Dieris B., Herpertz S. (2017). Suicidal ideation in adult patients with atopic dermatitis: A German cross-sectional study. Acta Derm. Venereol..

[17] Pompili M., Bonanni L., Gualtieri F., Trovini G., Persechino S., Baldessarini R.J. (2021). Suicidal risks with psoriasis and atopic dermatitis: Systematic review and meta-analysis. J. Psychosom Res..

[18] Sandhu J.K., Wu K.K., Bui T.L., Armstrong A.W. (2019). Association between Atopic Dermatitis and Suicidality: A Systematic Review and Meta-analysis. JAMA Dermatol..

[19] Cortés H., Rojas-Márquez M., Reyes-Hernández O.D., Morales-Morfín J.C., Guapillo-Vargas M.R.B., Varela-Cardoso M., Magaña J.J., Leyva-Gómez G., González-del Carmen M. (2021). Increased risk of depression and impairment in quality of life in patients with lamellar ichthyosis. Dermatol. Ther..

[20] Wang Y.-P., Gorenstein C. (2013). Psychometric properties of the Beck Depression Inventory-II: A comprehensive review. Rev. Bras Psiquiatr..

[21] Sanz J. (2014). Recomendaciones para la utilizacion de la adaptacion espanola del Inventario de Ansiedad de Beck (BAI) en la practica clinica. Clin. Salud.

[22] Hockberger R.S., Rothstein R.J. (1988). Assessment of suicide potential by nonpsychiatrists using the SAD PERSONS score. J. Emerg. Med..

[23] Chandramouleeswaran S., Edwin N.C., Victor P.J., Tharyan P. (2015). The emergency physician’s assessment of suicide risk in intentional self-poisoning using the modified SAD PERSONS scale versus standard psychiatric evaluation in a general hospital in South India: A cross-sectional study. Trop. Doct..

[24] Wu C.-Y., Huang H.-C., Wu S.-I., Sun F.-J., Huang C.-R., Liu S.-I. (2014). Validation of the Chinese SAD PERSONS Scale to predict repeated self-harm in emergency attendees in Taiwan. BMC Psychiatry.

[25] Kamalpour L., Rice Z.P., Pavlis M., Veledar E., Chen S.C. (2010). Reliable methods to evaluate the clinical severity of ichthyosis. Pediatr. Dermatol..

[26] Cortés H., Magaña J.J., Reyes-Hernández O.D., Zacaula-Juárez N., González-Torres M., Diaz-Beltrán W., León-Trejo M.C., Cariño-Calvo L., Leyva-Gómez G., Carmen M.G. (2019). Non-invasive analysis of skin mechanical properties in patients with lamellar ichthyosis. Skin Res. Technol..

[27] Cortés H., Rojas-Márquez M., Del Prado-Audelo M.L., Reyes-Hernández O.D., González-Del Carmen M., Leyva-Gómez G. (2022). Alterations in mental health and quality of life in patients with skin disorders: A narrative review. Int. J. Dermatol..

[28] Keefner T.P., Stenvig T. (2021). Suicidality: An Evolutionary Concept Analysis. Issues Ment. Health Nurs..

[29] Gupta M.A., Pur D.R., Vujcic B., Gupta A.K. (2017). Suicidal behaviors in the dermatology patient. Clin. Dermatol..

[30] Thyssen J.P., Hamann C.R., Linneberg A., Dantoft T.M., Skov L., Gislason G.H., Wu J.J., Egeberg A. (2018). Atopic dermatitis is associated with anxiety, depression, and suicidal ideation, but not with psychiatric hospitalization or suicide. Allergy Eur. J. Allergy Clin. Immunol..

[31] Rønnstad A.T.M., Halling-Overgaard A.S., Hamann C.R., Skov L., Egeberg A., Thyssen J.P. (2018). Association of atopic dermatitis with depression, anxiety, and suicidal ideation in children and adults: A systematic review and meta-analysis. J. Am. Acad. Dermatol..

[32] Kelly K.A., Balogh E.A., Kaplan S.G., Feldman S.R. (2021). Skin Disease in Children: Effects on Quality of Life, Stigmatization, Bullying, and Suicide Risk in Pediatric Acne, Atopic Dermatitis, and Psoriasis Patients. Child.

[33] Picardi A., Mazzotti E., Pasquini P. (2006). Prevalence and correlates of suicidal ideation among patients with skin disease. J. Am. Acad. Dermatol..

[34] Xu S., Zhu Y., Hu H., Liu X., Li L., Yang B., Wu W., Liang Z., Deng D. (2021). The analysis of acne increasing suicide risk. Medicine.

[35] Lukaviciute L., Navickas P., Navickas A., Grigaitiene J., Ganceviciene R., Zouboulis C.C. (2017). Quality of life, anxiety prevalence, depression symptomatology and suicidal ideation among acne patients in Lithuania. J. Eur. Acad. Dermatol. Venereol..

[36] Pavon Blanco A., Turner M.A., Petrof G., Weinman J. (2019). To what extent do disease severity and illness perceptions explain depression, anxiety and quality of life in hidradenitis suppurativa?. Br. J. Dermatol..

[37] Thorlacius L., Cohen A.D., Gislason G.H., Jemec G.B.E., Egeberg A. (2018). Increased Suicide Risk in Patients with Hidradenitis Suppurativa. J. Investig. Dermatol..

[38] Cavenagh A., Chatterjee S., Davies W. (2019). Behavioural and psychiatric phenotypes in female carriers of genetic mutations associated with X-linked ichthyosis. PLoS ONE.

[39] Lang U.E., Borgwardt S. (2013). Molecular mechanisms of depression: Perspectives on new treatment strategies. Cell Physiol. Biochem..

[40] Cortés H., Del Prado-Audelo M.L., Urbán-Morlán Z., Alcalá-Alcalá S., González-Torres M., Reyes-Hernández O.D., Carmen M.G.-D., Leyva-Gómez G. (2020). Pharmacological treatments for cutaneous manifestations of inherited ichthyoses. Arch. Dermatol. Res..

[41] Zaenglein A.L., Levy M.L., Stefanko N.S., Benjamin L.T., Bruckner A.L., Choate K., Craiglow B.G., DiGiovanna J.J., Eichenfield L.F., Elias P. (2021). Consensus recommendations for the use of retinoids in ichthyosis and other disorders of cornification in children and adolescents. Pediatr. Dermatol..

[42] Paljarvi T., McPherson T., Luciano S., Herttua K., Fazel S. (2022). Isotretinoin and adverse neuropsychiatric outcomes: Retrospective cohort study using routine data. Br. J. Dermatol..

[43] Abdelmaksoud A., Vojvodic A., Ayhan E., Dönmezdil S., Jovicevic T.V., Vojvodic P., Lotti T., Vestita M. (2019). Depression, isotretinoin, and folic acid: A practical review. Dermatol. Ther..

[44] Kim J., Kim J., Lee Y.I., Suk J., Lee D., Lee J.H. (2021). A pilot study evaluating the efficacy and safety of retinaldehyde-loaded niosomes against mild-to-moderate acne. J. Cosmet Dermatol..

[45] Liu J., Zheng A., Peng B., Xu Y., Zhang N. (2021). Size-Dependent Absorption through Stratum Corneum by Drug-Loaded Liposomes. Pharm. Res..

[46] Samadi A., Sartipi Z., Ahmad Nasrollahi S., Sheikholeslami B., Kashani M.N., Rouini M.R., Dinarvand R., Firooz A. (2022). Efficacy assessments of tretinoin-loaded nano lipid carriers in acne vulgaris: A double blind, split-face randomized clinical study. Arch. Dermatol. Res..

[47] Marathe K., Teng J.M.C., Guenthner S., Bunick C.G., Kempers S., Eads K., Castelo-Soccio L., Mendelsohn A.M., Raiz J., Murrell D.F. (2023). Topical Isotretinoin (TMB-001) Treatment for 12 Weeks Did Not Result in Clinically Relevant Laboratory Abnormalities in Participants with Congenital Ichthyosis in the Phase 2b CONTROL Study. Dermatol. Ther..

[48] Wincewicz K., Nasierowski T. (2020). Electrodermal activity and suicide risk assessment in patients with affective disorders. Psychiatr. Pol..

